# Prevalence of hepatitis B and C infection and linkage to care among patients with Non-Communicable Diseases in three rural Rwandan districts: a retrospective cross-sectional study

**DOI:** 10.1186/s12879-023-08678-y

**Published:** 2024-02-23

**Authors:** Tumusime Musafiri, Innocent Kamali, Casmille Kayihura, Jean de la Paix Gakuru, Francoise Nyirahabihirwe, Esdras Nizeyimana, Pilar Kandamage, Placide Habinshuti, Raymond Sekagarura, Jean Damascene Makuza, Nadine Karema, Janvier Serumondo, Theoneste Ntakirutimana, Jean d’Amour Ndahimana, Dale A. Barnhart

**Affiliations:** 1 Partners In Health/Inshuti Mu Buzima, Rwinkwavu, Rwanda; 2 Department of Public Health, Mount Kenya University, Kigali, Rwanda; 3 Ministry of Health, Butaro District Hospital Cancer Center of Excellence, Burera, Rwanda; 4 Rwanda Biomedical Centre, HIV, STIs, Viral Hepatitis and OVDC Division, Kigali, Rwanda; 5 School of Population and Public Health, University of British Columbia, Vancouver, BC Canada; 6 School of Public Health, College of Medecine and Health Sciences, University of Rwanda, Kigali, Rwanda; 7 Department of Global Health and Social Medicine, Harvard Medical School, Boston, MA USA; 8 Laterite Data, Research, and Analytics, Nairobi, Kenya

**Keywords:** Non-Communicable Diseases, Hepatitis B, Hepatitis C, Micro-elimination, Screening campaign, Rwanda

## Abstract

**Introduction:**

Rwanda’s Hepatitis C elimination campaign has relied on mass screening campaigns. An alternative “micro-elimination” strategy focused on specific populations, such as non-communicable disease (NCD) patients, could be a more efficient approach to identifying patients and linking them to care.

**Methods:**

This retrospective cross-sectional study used routine data collected during a targeted screening campaign among NCD patients in Kirehe, Kayonza, and Burera districts of Rwanda and patients receiving oncology services from the Butaro District Hospital. The campaign used rapid diagnostic tests to screen for Hepatitis B surface antigen (HBsAg) and Hepatitis C antibody (anti-HCV). We reported prevalences and 95% confidence intervals for HBsAg and anti-HCV, assessed for associations between patients’ clinical programs and hepatitis B and C, and reported cascade of care for the two diseases.

**Results:**

Out of 7,603 NCD patients, 3398 (45.9%) self-reported a prior hepatitis screening. Prevalence of HBsAg was 2.0% (95% CI: 1.7%-2.3%) and anti-HCV was 6.7% (95% CI: 6.2%-7.3%). The prevalence of HBsAg was significantly higher among patients < 40 years (2.4%). Increased age was significantly associated with anti-HCV (12.0% among patients ≥ 70 years). Of the 148 individuals who screened positive for HbsAg, 123 had viral load results returned, 101 had detectable viral loads (median viral load: 451 UI/mL), and 12 were linked to care. Of the 507 individuals who screened positive for anti-HCV, 468 had their viral load results returned (median viral load: 1,130,000 UI/mL), 304 had detectable viral loads, and 230 were linked to care.

**Conclusion:**

Anti-HCV prevalence among Rwandan patients with NCD was high, likely due to their older age. NCD-HCV co-infected patients had high HCV viral loads and may be at risk of poor outcomes from hepatitis C. Hepatitis C micro-elimination campaigns among NCD patients are a feasible and acceptable strategy to enhance case detection in this high-prevalence population with elevated viral loads and may support linkage to care for hepatitis C among elderly populations.

## Introduction

Viral hepatitis causes over one hundred thousand deaths per year in sub-Saharan Africa [1]. Effective treatment for hepatitis B (HBV) and a functional cure for hepatitis C (HCV) are associated with decreased rates of mortality, cirrhosis, hepatic decompensation, and hepatocellular carcinoma, as well as improved production and quality of life [2]. The estimates for the global prevalence of HBV and HCV are 3.5% and 2.8%, respectively, [3] compared to 2.5% to 2.9% in Africa [4]. For HCV in particular, the recent discovery of direct acting antiretroviral (DAAs) provides a new highly efficacious treatment option that can be successfully delivered in low-resource settings [5]. For HBV, available treatments are categorized into 2 classes including interferons (standard interferon-α-2b, peginterferon-α-2a and nucleoside or nucleotide analogues (lamivudine, adefovir, entecavir, telbivudine, tenofovir disoproxil fumarate, and tenofovir alafenamide), which are effective in suppressing HBV replication but do not eradicate the virus [6, 7]. Despite the availability of treatments for both HBV and HCV, most people remain unaware of their hepatitis status until symptoms appear, resulting in liver damage and poor health outcomes [8].

In light of the burden of viral hepatitis and the availability of effective treatment options, Rwanda launched a 5-year strategic plan for HCV elimination by 2024 [9]. This plan, which involves both case identification and treatment of identified cases, relies heavily on providing widespread testing for both HBV and HCV, which has largely been conducted through mass screening campaigns that target the general population [10, 11]. However, HCV elimination strategies that use mass screening approaches among the general population is daunting, complex and highly priced with cost estimates running in the billions of euros of in high-income countries [12] and 45 million dollars in Rwanda along [13], and billions of euros One alternative approach is a “micro-elimination” strategy, which breaks down national elimination goals into smaller, achievable goals within specific segments of the population so that targets can be achieved in a shorter period of time with fewer financial resources [14, 15].

One potential target population for hepatitis micro-elimination is non-communicable disease (NCD) patients. These patients may be at elevated risk for HCV due to their older average age, which is a risk factor that is associated with HCV and with many common NCDs [16, 17]. For example, a recent study in Butaro found that the prevalence of HCV infection was found to be 9.4% among individuals aged 45 and above [18]. Additionally, some studies suggest that chronic HCV infection can cause extrahepatic conditions, such as type 2 diabetes, Circulatory diseases, and Chronic kidney diseases (CKD) [19–21]. Furthermore, because NCD patients are already in regular contact with the healthcare system, they may be more easily mobilized for screening and linkage to treatment compared to other high-risk populations.

Although Rwanda has conducted limited targeted screening among people living with human immunodeficiency virus (HIV), pregnant women [10, 22], prisoners, sex workers, injection drug users, and men who have sex with men (MSM) [23], NCD patients are not specifically targeted for hepatitis screening by the national Rwandan hepatitis program. In this context, our team implemented a targeted HBV and HCV screening among patients with NCDs in rural setting. This paper reports on the sero-prevalence and associated risk factors for HBsAg and anti-HCV among NCD patients in three rural Rwandan districts as well as the cascade of care and viral load results observed among patients who screened positive.

## Methods

### Study design

This study is a retrospective cross-sectional study used routine data collected during the screening of HBV and HCV among NCD patients in three rural districts of Rwanda.

### Setting

The screening program was led by Partners In Health/Inshuti Mu Buzima (PIH/IMB), an international non-government organization that supports health care implementation in three rural Rwandan districts (Kirehe, Kayonza and Burera) in partnership with the Rwandan Ministry of Health. PIH/IMB supports Kirehe, Rwinkwavu, and Butaro district hospitals together with their 44 affiliated health centers. The screening campaign were conducted from November 2020 to March 2021.

### Participants

All NCD patients aged 15 years and above receiving care at health facilities within the Kirehe, Butaro and Rwinkwavu(in Kayonza) District Hospitals’catchment areas were invited to participate in a voluntary mass screening campaign for HBV and HCV. In accordance with national guidelines for hepatitis screening and treatment [24], patients were excluded from screening if they were under 15 years of age or if they had already been initiated on treatment for HBV or HCV. However, some patients who had already been initiated on treatment were provided with viral load testing if the timing of the screening aligned with clinical guidelines for viral load monitoring [24]. Oncology patients who received out-patient or in-patient oncology services from the Butaro District Hospital Cancer Center of Excellence during the screening campaign were also eligible to participate. However, because the Cancer Center serves patients from around the country, and some of them often travel long distances to the Cancer Center, oncology patients were not invited to attend screening unless a screening day coincided with an existing appointment. Non-NCD patients at the health center, such as patients receiving care for mental health, were provided with screening on request in the interest of furthering the national elimination targets and providing equitable access to healthcare, but were not explicitly invited to participate.

### Screening campaign

Community awareness for the campaign was initially done through meetings with local leaders, the director general from each district hospital, the heads of health centers, and local community health workers. In collaboration with the NCD program of PIH/IMB, a list of NCD patients attending each health facility was generated and patients were informed of the date when the campaign would come to their nearest health facility via telephone or community health worker.

Prior to testing, we conducted a group education, information, and communication session regarding viral hepatitis and obtained verbal consent for HBV and HCV screening. During screening, trained nurses and laboratory technicians collected capillary blood samples to test for anti-HCV and HBsAg using SD Bioline rapid diagnostic tests (RDTs). These tests are among rapid diagnosis tests that are pre-approved by World Health Organization(WHO) and are manufactured by Abbott Diagnostics Korea Inc, Giheung-gu, Korea [25, 26], and have a sensitivity and specificity of > 99% to detect anti-HCV and sensitivity of 96.7% and specificity of 98.9% to detect HBsAg [27]. For patients who screened positive, 4-5 ml of blood for HBV Deoxyribonucleic Acid (DNA) and/or HCV Ribonucleic Acid (RNA) was drawn from each participant through venipuncture using a vacutainer and an ethylene-diamine-tetra-acetic acid (EDTA) anticoagulant tube for viral load testing.

The collected blood samples from health facilities in Kirehe and Kayonza catchment areas were transported to Kirehe District Hospital every day, while those from Burera District were transported to Ruhengeri district hospital for viral load testing. These two hospitals are among the closest district hospitals in the country that have the necessary equipment for hepatitis viral load testing. During transportation, samples were placed in the tube rack, and put into cooler box for triple packing as recommended [28, 29]. Blood samples were centrifuged (Universal 320 R) at 3000 rpm to separate plasma from whole blood and plasma was used for viral load testing. If the plasma was not analyzed on the same day, it was kept at -20^0^C until analysis is done. Viral load testing was performed using COBAS 480 HCV and HBV Test, V.2.0: Quantitative (Roche) with a lower limit of quantification of 15 IU/mL and 10 IU/Ml for HCV and HBV, respectively.

#### Variables & data sources

This analysis used secondary data that were collected during patient registration, screening, and viral load testing. During the screening campaign, data was recorded into Research Electronic Data Capture (REDCap) database for operational purposes and used to facilitate patient follow-up.

Social demographic information was collected during patient registration process using a digitalized form programmed into REDCap. Before analysis, duplicate patient entries and patient who had previously screened positive and were already on treatment for hepatitis were removed from analysis. Names, identifying numbers, and address were removed from the dataset before analysis to maintain patient health records confidentiality.

#### Study size

The targeted screening campaign was expected to cover 9,920 NCD patients as well as any oncology patients receiving out-patient or in-patient oncology services from the Butaro District Hospital Cancer Center of Excellence during the screening campaign. Of the 8,125 individuals who attended the targeted screening campaign, 7,622 were NCD patients, reflecting a screening coverage of 76.7% among NCD patients, 160 were oncology patients, and 344 were other visitors at the health center who requested a screening.

#### Statistical methods and data analysis

We described demographic characteristics of patients using percentages and frequencies. We calculated prevalence and 95% confidence intervals for HBsAg and anti-HCV in the overall population of patients who participated in the screening campaign. We assessed bivariate associations between clinical NCD program and prevalence of HBsAg and anti-HCV using Fisher’s exact or Chi-squared tests. Because age and sex are known to be strong risk factors for viral hepatitis in Rwanda, we used multivariate logistic regressions to determine the relationship between the demographic characteristics and HBsAg and anti-HCV after adjusting for age and sex.

To understand whether patients with NCD were at higher risk of hepatitis compared to the general population, we also compared prevalence among NCD patients with the recent results from the nationally-representative Rwanda Population-Based HIV Impact Assessment(RPHIA) [30], which published prevalence of HBsAg and anti-HCV among Rwandans aged 15–64. We calculated the crude prevalence of HBsAg and anti-HCV among the subset of NCD patients who were 15–65 years of age. We also directly standardized to the age distribution of RPHIA survey respondents using 5-year age categories. This directly standardized prevalence can be interpreted as the expected prevalence that we would have observed if the NCD patients had the same distribution of ages as the RPHIA participants. The age distribution of our screening participants as well as the reference population is given Table 5 in Appendix 1.

Among patients who screened positive, we also describe the cascade of care by reporting the counts and frequencies of patients who had their viral load results returned, had detectable viral loads, and were linked to care. For patients with detectable viral loads (102 for HBV and 304 for HCV), we reported the median and interquartile ranges (IQR) for viral loads.

## Results

Of the 8,125 individuals who participated in screening, we excluded 198 patients with a prior diagnosis of hepatitis C and 324 additional patients who did not belong to the NCD or oncology program leaving a total of 7,603 screening participants to be included in analysis. Half of all participants were from Burera district (*n* = 3813, 50.2%), a third from Kirehe district (*n* = 2326, 30.6%), and the remaining from Kayonza District (*n* = 1460, 19.2%), (Table 1). The majority of NCD patients were female (*n* = 5,978, 78.7%), over 60 years (*n* = 4488, 59.8%), were married or cohabitating (*n* = 4752, 62.7%), and had received less than a primary education (*n* = 6032, 81.4%). The plurality was in Ubudehe 3 or 4 (*n* = 3,077, 41.0%) and the overwhelming majority of participants were using community-based health insurance (CBHI) commonly called Mutuelle (*n* = 7,358; 97.9%). Patients were most likely to be enrolled in the NCD program for hypertension (*n* = 6032, 81.4%) almost half of participants self-reported being previously screened for hepatitis (*n* = 3398, 45.9%) (Table1).

**Table 1:** Demographic characteristics of screening participants (*N* = 7,603)

	**N**	**%**
**District (*n* = 7,599)**		
Burera	3813	50.2%
Kayonza	1460	19.2%
Kirehe	2326	30.6%
**Age (*n* = 7,505)**		
< = 39	542	7.2%
40 to 49	938	12.5%
50 to 59	1537	20.5%
60 to 69	2214	29.5%
> = 70	2274	30.3%
**Sex (*n* = 7,598)**		
Female	5978	78.7%
Male	1620	21.3%
**Marital status (*n* = 7,573)**		
Single	210	2.8%
Married/Cohabitating	4752	62.7%
Widowed	2446	32.3%
Divorced	165	2.2%
**Insurance (*n* = 7,518**)		
Mutuelle	7358	97.9%
Private insurance	118	1.6%
No insurance	42	0.6%
**Ubudehe (*n* = 7,505)**		
Category 1	1873	25.0%
Category 2	2555	34.0%
Category 3 or 4	3077	41.0%
**Education (*n* = 7,412)**		
Did not complete primary school	6032	81.4%
Completed primary or higher	1380	18.6%
**Patient’s clinical programs**^a^		
Hypertension	6099	80.2%
Chronic respiratory disease or asthma	1080	14.2%
Diabetes	517	6.8%
Heart failure	220	2.9%
Oncology	157	2.1%
Kidney disease	57	0.7%
**Self-reported prior hepatitis screening (*n* = 7402)**	3398	45.9%

^a^Patients could be registered in more than one clinical program

Prevalence of HBsAg was 2.0% (95% CI: 1.7%-2.3%) and anti-HCV was 6.7% (95% CI: 6.2%-7.3%). HBsAg was not associated with any clinical program in either the crude of adjusted analyses (Table 2). Anti-HCV prevalence was higher in the hypertensive patients compare to other NCD programs (6.6% vs 5.7%), but this difference was not statistically significant after adjusting for age and sex (OR = 1.1, 95% CI: 0.9, 1.5, *p* = 0.358 (Table 2).

**Table 2:** Association between patient’s characteristics and hepatitis B surface antigen (HBsAg), and hepatitis C antibody (anti-HCV), *N* = 7,463^a^

Variable	Hepatitis B								Hepatitis C							
	**HBsAg positive**		**HBsAg Negative**			***Adjusted Odds Ratio***^b^			**Anti-HCV positive**		**Anti-HCV negative**			***Adjusted Odds Ratio***^b^		
	**N**	**%**	**N**	**%**	***p-value***	***OR***	***95% CI***	***p-value***	**N**	**%**	**N**	**%**	***p-value***	***OR***	***95% CI***	***p-value***
**District (*n* = 7,461)**					*0.515*	*0.455*							*0.711*			0.147
Butaro	70	1.9%	3,689	98.1%		*ref*	*---*	*---*	235	6.3	3,505	93.7		*ref*	*---*	*---*
Kayonza	20	1.4%	1409	98.6%		*0.7*	*(0.4, 1.2)*	*---*	90	6.2	1,357	93.8		*1.1*	*(0.9, 1.5)*	*---*
Kirehe	41	1.8%	2232	98.2%		*0.9*	*(0.6, 1.4)*	*---*	154	6.8	2,120	93.2		*1.2*	*(1.0, 1.5)*	*---*
**Age**					*0.145*	*0.165*							** < *0.001***			** < *0.001***
< = 39	13	2.4%	527	97.6%		*ref*	*---*	*---*	5	0.9	535	99.1		*ref*	*---*	*---*
40 to 49	23	2.5%	907	97.5%		*1*	*(0.5, 2.1)*	*---*	11	1.2	925	98.8		*1.3*	*(0.4, 3.7)*	*---*
50 to 59	30	2.0%	1502	98.0%		*0.8*	*(0.4, 1.60*	*---*	39	2.5	1,496	97.5		*2.8*	*(1.1, 7.2)*	*---*
60 to 69	31	1.4%	2169	98.6%		*0.6*	*(0.3, 1.1)*	*---*	134	6.1	2,068	93.9		*7*	*(2.8, 17.1)*	*---*
> = 70	34	1.5%	2227	98.5%		*0.6*	*(0.3, 1.2)*	*---*	290	12.9	1,960	87.1		*16*	*(6.5, 38.8)*	*---*
**Sex**					*0.031*	*0.033*							*0.233*			*0.216*
Female	93	1.6%	5786	98.4%		*ref*	*---*	*---*	367	6.2	5.512	93.8		*ref*	*---*	*---*
Male	38	2.4%	1546	97.6%		*1.5*	*(1.0, 2.2)*	*---*	112	7.1	1,472	92.9		*1.2*	*(0.9, 1.4)*	*–-*
**Marital status (*n* = 7,438)**					*0.207*			*0.774*					** < *0.001***			*0.460*
Single	6	2.9%	202	97.1%		*ref*	*---*	*---*	5	2.4	202	97.6		*ref*	*---*	*---*
Married/Cohabitating	89	1.9%	4582	98.1%		*0.7*	*(0.3, 1.7)*	*---*	261	5.6	4,410	94.4		*1.6*	*(0.6, 3.9)*	*---*
Widowed	33	1.4%	2364	98.6%		*0.6*	*(0.2, 1.7)*	*---*	205	8.6	2,190	91.4		*1.5*	*(0.6, 3.9)*	*---*
Divorced	2	1.2%	160	98.8%		*0.5*	*(0.1, 2.5)*	*---*	5	3.0	160	97.0		*0.8*	*(0.2, 3.0)*	*---*
**Insurance (*n* = 7,439)**					*0.869*	*0.355*							*0.011*			*0.114*
Mutuelle	129	1.8%	7151	98.2%		*ref*	*---*	*---*	452	6.22	6,815	93.8		*ref*	*---*	*---*
Private insurance	1	0.9%	116	99.2%		*0.4*	*(0.1, 2.8)*	*---*	2	1.74	113	98.3		*0.5*	*(0.1, 2.0)*	*---*
No insurance	0	100.0%	42	100.0%		*–*	*---*	*-4*	6	14.6	35	85.4		*2.3*	*(0.9, 5.6)*	*---*
**Ubudehe (*n* = 7197)**					*0.779*	*0.756*							** < *0.001***			*0.0868*
Category 1	32	1.7%	1814	98.3%		*ref*	*---*	*---*	167	9.0	1,680	91.0		*ref*	*---*	*---*
Category 2	40	1.6%	2478	98.4%		*0.8*	*(0.5, 1.3)*	*---*	153	6.1	2,363	93.9		*0.9*	*(0.7, 1.1)*	*---*
Category 3 & 4	56	1.8%	2994	98.2%		*0.9*	*(0.6, 1.5)*	*---*	153	5.0	2,894	95.0		*0.8*	*(0.6, 1.0)*	*---*
**Education (*n* = 7660)**					*0.831*	*0.249*							** < *0.001***			*0.061*
Less than primary	96	1.6%	5869	98.4%		*ref*	*---*	*---*	356	6.0	5,595	94.0		*ref*	*---*	*---*
Primary or higher	21	1.5%	1352	98.5%		*0.7*	*(0.5, 1.2)*	*---*	34	2.5	1,338	97.5		*0.7*	*(0.5, 1.0)*	*---*
**Hypertension**					*0.264*	*0.741*							** < *0.001***			*0.358*
No	31	2.1	1,447	97.9		*ref*	*---*	***---***	63	4.3	1,417	95.7		*ref*	*---*	*---*
Yes	100	1.7	5,885	98.3		*0.9*	*(0.6, 1.4)*	*–-*	416	7.0	5,567	93.1		*1.1*	*(0.9, 1.5)*	*---*
**Chronic respiratory/asthma**					*0.267*	*0.537*							*0.27*			*0.636*
No	108	1.7	6,295	98.3		*ref*	*---*	*---*	419	6.6	5,982	93.5		*ref*	*---*	*---*
Yes	23	2.2	1,037	97.8		*1.2*	*(0.7, 1.8)*	*---*	60	5.7	1,002	94.4		*1.1*	*(0.8, 1.4)*	*---*
**Diabetes**					*0.073*	*0.183*							*0.22*			*0.658*
No	117	1.7	6,840	98.3		*ref*	*---*	*---*	453	6.5	6,503	93.5		*ref*	---	*---*
Yes	14	2.8	492	97.2		*1.5*	*(0.8, 2.6)*	*---*	26	5.1	481	94.9		*1.1*	*(0.7, 1.7)*	*---*
**Heart Failure**					*0.25*	*0.304*							*0.547*			*0.512*
No	125	1.7	7,121	98.3		*ref*	*---*	*---*	463	6.4	6,784	93.6		*ref*	*---*	*---*
Yes	6	2.8	211	97.2		*1.5*	*(0.7, 3.6)*	*---*	16	7.4	200	92.6		*1.2*	*(0.7, 2.0)*	*–-*
**Oncology**					> *0.99*	*0.534*							*0.004*			*0.082*
No	129	1.77	7,178	98.2		*ref*	*---*	*---*	477	6.5	6,831	93.5		*ref*	*---*	*---*
Yes	2	1.28	154	98.7		*0.6*	*(0.2, 2.6)*	*---*	2	1.3	153	98.7		*0.3*	*(0.1, 1.2)*	*---*
**Kidney Disease**					*0.627*								*0.784*			*0.786*
No	131	1.8	7,276	98.2		*ref*	*---*	*---*	475	6.4	6,931	93.6		*ref*	*---*	*---*
Yes	0	0	56	100		^*---*c^	*---*	*---*	4	7.0	53	93.0		*1.2*	*(0.4, 3.3)*	*---*
**Self-reported prior screened for Hep B or Hep C (*n* = 7,330)**					*0.271*	*0.346*							*0.191*	*0.602*		
No	59	1.5	3,909	98.5		*ref*	*---*	*---*	255	6.4	3,708	93.6		*ref*	*---*	*---*
Yes	61	1.8	3,301	98.2		*1.2*	*(0.8, 1.7)*	*---*	191	5.7	3,160	94.3		*1.1*	*(0.9, 1.3)*	*---*

^a^Sample size unless otherwise indicated. Excludes 140 participants who are missing data on rapid diagnostic test for hepatitis B, and hepatitis C viral load, age, or sex

^b^Odds ratio estimated from a logistic regression that includes age and sex

^c^Odds ratio not estimated due to lack of cases among those with kidney disease

When we restricted our sample to ages 15–64 and directly standardized our age distribution to the ages of respondents to the Rwandan Population-based HIV Impact Assessment 2018–2019, we observed that, after adjusting for age, NCD patients presented similar risks of anti-HBsAg and anti-HCV compared to the general population (Table 3).

**Table 3:** Prevalence of HBsAg and anti-HCV among NCD screening participants aged 15–64 (*N* = 4,459) compared to age-standardized prevalence in the general population

	Prevalence among NCD screening participants aged 15–64 years		^a^Prevalence from RPHIA participants aged 15–64
Infection by Sex	Crude prevalence and 95% CI	^a^Directly standardized to the age distribution of the Rwandan Population Based HIV Impact Assessment 2018–2019	
HBsAg			
Female	1.8% (1.3%, 2.3%)	1.7%	1.3%
Male	3.3% (2.2%, 4.7%)	2.4%	2.8%
Total	2.1% (1.7%, 2.6%)	1.8%	2.0%
Anti-HCV			
Female	2.7% (2.2%, 3.3%)	1.0%	1.1%
Male	2.6% (1.7%, 3.9%)	0.9%	1.3%
Total	2.7% (2.2%, 3.2%)	1.0%	1.2%

^a^Data on the age distribution and age- and sex-specific hepatitis prevalence extracted from the Rwandan Population- base HIV Impact Assessment 2018–2019

**Fig. 1:**
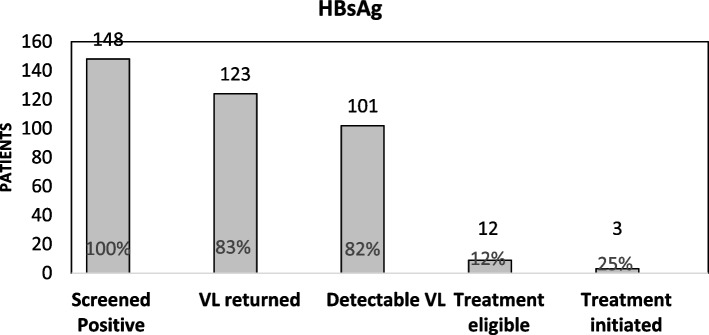
Cascade of Care for HBsAg

**Fig. 2:**
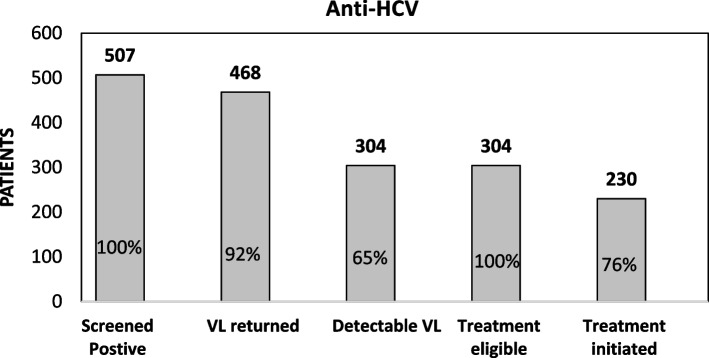
Cascade of Care for Ant-HCV. Cascade of care for management of hepatitis C among NCD patients identified in the mass screening campaign

Of the 148 individuals who screened positive for HbsAg, 123 (83%) had their viral load results returned and recorded, 101 (82%) of these had detectable viral loads, and 12 (12%) successfully linked to care (Fig. 1). Median detectable viral load among individuals with detectable HBV was 451 UI/mL (IQR: 166–1860) (Table 4). Of the 507 individuals who screened positive for anti-HCV, 468 (92%) had their HCV viral load results returned and recorded, 304 of these (65%) had detectable viral loads and were eligible for treatment, and 230 (76%) were linked to care (Fig. 2). Median detectable viral load among individuals with detectable hepatitis C viral load was 1,130,000 UI/mL (IQR: 323,000–2,700,000) (Table 4).

**Table 4:** Viral load results among NCD patients who screened positive of Hepatitis B surface antigen (HBsAg) or Hepatitis C antibody (anti-HCV)

Variable	Detectable viral load N(%)	Median number of detectable viral Load	IQR
HBV DNA	102 (82.3%)	451	166–1860
HCV RNA	304(65.0%)	1,130,000	323,000–2,700,000

## Discussion

In this study, we examined HBsAg and anti-HCV sero-prevalences. We assessed risk factors associated with HBsAg and Anti-HCV among NCD and oncology patients participating in a targeted screening campaign in three rural Rwandan districts. Overall, HBsAg prevalence among NCD patients who participated in our screening campaign (2.0%) was comparable to previous reports from RPHIA 2.0% [30]. Similar to the findings of other studies conducted in Rwanda [18, 31], our research revealed a potential higher risk of HBV infection among males in our study population when compared to females.

The overall prevalence of anti-HCV among NCD patients was 6.7%, which is much higher than the 1.2% prevalence of anti-HCV reported in RPHIA. These finding likely stem from the strong association between age and hepatitis C, which has been repeatedly found to be a major risk factor for anti-HCV [16, 17, 32]. Thirty percent of patients who participated in this targeted NCD screening campaign were over the age of 70, making them meaningfully older than both the general population in Rwanda and the RPHIA respondents.

After standardizing to account for the advanced age of the NCD patients, we found that the risk of HBsAg among NCD patients compared to the general population was slightly lower overall (1.8% vs. 2.0%) and among men (2.4% vs. 2.8%) but slightly higher among women (1.7% vs. 1.3%). NCD patients were at slightly lower risk of anti-HCV overall (1.0 vs. 1.2%), among men (0.9% vs. 1.3%) and among women (1.0% vs. 1.1%). Similarly, despite the fact that A. Lecube et al., [19], and M. Basaranoglu et al., [33] have previously reported that HCV infection is associated with diabetes mellitus, we did not find any evidence that any NCD diagnosis was associated with hepatitis after adjusting for age and sex. Collectively, these findings suggest that NCD patients face an elevated risk of HCV compared to other Rwandans due to their advanced age rather than due to other NCD-related factors or individual behaviors.

While the risk of HCV among NCD patients in Rwanda is similar to among other elderly Rwandan populations [18, 34, 35], the high overall prevalence of HCV means that, they are still strong candidates for a targeted screening campaign. Furthermore, there is some evidence to suggest that the NCD patients in our campaign may be at elevated risk of poor health outcomes from HCV. Although the proportion of anti-HCV positive patients with a detectable viral load was lower (65%) than what has been reported elsewhere in Rwanda (78%) [36], the median hepatitis C viral load among NCD patients observed in this study was high (1,130,000UI/ML). Moucar R et al., [37] conducted a study that demonstrated that Insulin Resistance was associated with a higher serum HCV RNA level. This suggests that there may be biological mechanisms that make NCD-HCV co-infected patients more vulnerable to high HCV viral loads and more susceptible to subsequent poor health outcomes compared to the patients with HCV alone.

Our study also shows that almost half of NCD patients self-reported prior hepatitis screening. However, a history of previous screening was not associated with a reduced risk of disease. This pattern of repeated screening and prevalent hepatitis among repeat screeners suggests a suboptimal linkage to hepatitis treatment. Furthermore, during our campaign, many NCD patients reported having been previously diagnosed with hepatitis infection, but they often did not know which type of hepatitis infection they had. This lack of knowledge may prevent patients from accessing appropriate care, as has been discussed previously [38]. Investing in high-quality electronic data collection during screening campaigns, using mobile hepatitis treatment clinics to provide decentralized care, a three strategies that we have used to successfully mitigate those problems [36, 39]. However, even within our targeted screening campaign, there were substantial gaps in linkage to care, particularly among NCD patients who screened positive for HBsAg. Our previous research has identified that incomplete collection of patient contact information, delays in turn-around times for viral load testing, and costs associated with accessing pre-treatment-initiation lab tests are important barriers to linking patients to timely treatment for viral hepatitis [36, 40]. Embedding hepatitis screening campaigns within existing chronic care programs, such as the NCD program, may mitigate some of these challenges because the patients have existing relationships and repeated points of contact with the health care system. However, investing in more efficient laboratory processes, and healthcare provider training will be necessary to ensure that access to hepatitis diagnosis, and treatment are truly accessible.

Our work does have some limitations. First, our analysis relies on routine data collected during the screening campaign, and there was no comprehensive questionnaire to collect information on all known risk factors for hepatitis. This routine data suffers from some inaccuracies or missingness, particularly around the return of viral load results and linkage to care. Second, this study was conducted within the catchment areas of three rural Inshuti Mu Buzima (IMB)-supported Rwandan district hospitals and as such may have limited generalizability. However, given that most district hospitals in Rwanda and in the region are located in rural settings with similar population characteristics, these results can likely be extrapolated to other similar settings in Rwanda or elsewhere.

## Conclusion

Our findings do suggest that NCD patients are good target populations for hepatitis C micro-eliminiation campagins in Rwanda. First, most NCD patients are at elevated risk of hepatitis C due to their advanced age. Second, they may suffer more severe outcomes from hepatitis due to higher viral loads among NCD-HCV co-infected patients. Third, in settings where linkage to care among the general population remains challenging, targeting NCD patients who already have established connections to the healthcare system appears to be an acceptable opportunity for mobilizing the community and may enhance linkage to care for elderly patients. While NCD patients are not at higher risk of HBV, including HBV screening and linkage to treatment as part of HCV elimination campaigns is both feasible and acceptable. Because increased age is associated with HCV in many settings, the micro-elimination of HCV among NCD pateints is a strategy that could be considered in a wide variety of settings.

## Data Availability

The datasets used and/or analyzed during the current study are available from the corresponding author or the Inshuti Mu Buzima Research committee (imbrc@pih.org) on reasonable request.

## Appendix 1

Table 5.

**Table 5:** Age distribution of screening participants aged 15–64 and the reference age distribution used for direct standardization

	**Observed age distribution of screening participants**			**Age distribution of reference population (RPHIA)** ^a^
	**Women**	**Men**	**Total**	
15–19	0%	1%	0%	18%
20–24	1%	1%	1%	14%
25–29	1%	3%	1%	13%
30–34	3%	3%	3%	13%
35–39	7%	7%	7%	12%
40–44	10%	11%	10%	9%
45–49	12%	12%	12%	6%
50–54	16%	15%	16%	6%
55–59	22%	20%	21%	6%
60–64	29%	26%	28%	4%

^a^Data on the age distribution and age- and sex-specific hepatitis prevalence extracted from the Rwandan Population- base HIV Impact Assessment 2018–2019.

## Abbreviations

CBHI: Community Health Insurance
CI: Confidence Interval
DAAs: Direct Acting Antivirals
HCV: Hepatitis C Virus
IHDPC: Institute of HIV Disease Prevention and Control
IMBRC: Inshuti Mu Buzima Research Committee
IQR: Interquartile range
PIH/IMB: Partners In Health/Inshuti Mu Buzima
LMIC: Low and Middle Income Countries
MoH: Ministry of Health
SVR12: Sustained Virologic Response after 12 weeks
RNEC: Rwanda National Ethics Committee
RDT: Rapid Diagnostic Test
RBC: Rwanda Biomedical Centre; REDCap: Research Electronic Data Capture
RNA: Ribonucleic Acid
WHO: World Health Organization
HBsAg: Antibody to Hepatitis B surface antigen
HBV: Hepatitis B Virus
HCVab: Hepatitis C Virus antibody
DNA: Deoxyribonucleic acid
HIV: Human Immunodeficiency Virus
NCDs: Non-Communicable Diseases
RPHIA: Rwanda Population-Based HIV Impact Assessment
MSM: Men who have Sex with Men
REDCap: Research Electronic Data Capture
